# O028: Impact of a prevention and control infection program in a tertiary care teaching hospital

**DOI:** 10.1186/2047-2994-2-S1-O28

**Published:** 2013-06-20

**Authors:** RE Quirós, L Fabbro, A Novau, G Kremer, M Casanova, M Pereyra

**Affiliations:** 1 Prevention and Infection Control Department, Hospital Universitario Austral, Caba, Argentina

## Introduction

The implementation of an expanded surveillance system of healthcare-associated infections (HAIs) at institutional level is necessary since these events can occur outside of intensive care units. In addition, this comprehensive information allows evaluating the impact of the Prevention and Control Infection Program (PCIP) in terms of reduction of HAIs.

## Objectives

The aim of this study was to estimate the net savings associated to the implementation of a PCIP at a tertiary care teaching hospital.

## Methods

Since Jan’10 different components of PCIP were progressively implemented (Hand hygiene program, Prevention of emergency and transmission of multidrug resistant microorganisms through isolation measures, environmental cleaning and antimicrobial stewardship, Care bundles for the prevention of device associated infections, and a Prevention program for surgical site infection). According to this strategy since Jul’10 an expanded surveillance system using National Healthcare Safety Network (NHSN) methodology was implemented. In order to evaluate the impact of PICP, the HAIs avoided were estimated by comparing infection rates, as events per 1000 patient-days, of two years (2011 vs 2012). All costs are expressed in US dollars. The local attributable cost of HAIs was estimated by adjusting the present value of previously reported data[1]. The cost of PCIP was estimated from the incremental costs associated with human resources and the strategies implemented as part of the PCIP. Finally, the net savings was estimated as a difference between the incremental costs and direct costs avoided.

## Results

The rate of HAIs in 2011 was 14.17 episodes per ‰ patient-days in comparison with 10.00 episodes per ‰ patient-days in 2012 (difference 4.17; CI 95% 2.73 to 5.62; p<0.0001) with 188 HAIs avoided after adjusting by patient-days of 2012. While the gross saving associated with these episodes was $100,730, the overall net saving was $320,421.

## Conclusion

A comprehensive PCIP was a cost-effective strategy to reduce the incidence of HAIs in our institution. This type of analysis is a useful tool when negotiating additional resources with managers.

## Disclosure of interest

None declared.

## References

[B1] Quirós RE Impact of nosocomial infections in Argentina: net cost associated with implementing effective infection control programs 5 th Decennial ICHAI 2010 Atlanta

